# Pay More Attention: a national mixed methods study to identify the barriers and facilitators to ensuring equal access to high-quality hospital care and services for children and young people with and without learning disabilities and their families

**DOI:** 10.1136/bmjopen-2016-012333

**Published:** 2016-12-09

**Authors:** Kate Oulton, Jo Wray, Lucinda Carr, Angela Hassiotis, Carey Jewitt, Sam Kerry, Irene Tuffrey-Wijne, Faith Gibson

**Affiliations:** 1 Centre for Outcomes and Experience Research in Children's Health, Illness and Disability (ORCHID), Great Ormond Street Hospital NHS Foundation Trust, London, UK; 2 JM Barrie Division, Great Ormond Street Hospital NHS Foundation Trust, London, UK; 3 Division of Psychiatry, UCL, London, UK; 4 UCL Knowledge Lab, UCL Institute of Education, London, UK; 5 Kingston University and St George's, University of London, London, UK; 6 School of Health Science, University of Surrey, Surrey, UK

**Keywords:** Intellectual Disability, Mixed methods, PAEDIATRICS

## Abstract

**Introduction:**

Despite evidence of health inequalities for adults with intellectual disability (ID) there has yet to be a comprehensive review of how well hospital services are meeting the needs of children and young people (CYP) with ID and their families. We do not know how relevant existing recommendations and guidelines are to CYP, whether these are being applied in the paediatric setting or what difference they are making. Evidence of parental dissatisfaction with the quality, safety and accessibility of hospital care for CYP with ID exists. However, the extent to which their experience differs from parents of CYP without ID is not known and the views and experiences of CYP with ID have not been investigated. We will compare how services are delivered to, and experienced by CYP aged 5–15 years with and without ID and their families to see what inequalities exist, for whom, why and under what circumstances.

**Methods and analysis:**

We will use a transformative, mixed methods case study design to collect data over four consecutive phases. We will involve CYP, parents and hospital staff using a range of methods; interviews, parental electronic diary, hospital and community staff questionnaire, patient and parent satisfaction questionnaire, content analysis of hospital documents and a retrospective mapping of patient hospital activity. Qualitative data will be managed and analysed using NVivo and quantitative data will be analysed using parametric and non-parametric descriptive statistics.

**Ethics and dissemination:**

The study will run from December 2015 to November 2018. We have Health Authority Approval (IRAS project ID: 193932) for phase 1 involving staff only and ethical and Health Authority Approval for phases 2–4 (IRAS project ID: 178525). We will disseminate widely to relevant stakeholders, using a range of accessible formats, including social media. We will publish in international peer-reviewed journals and present to professional, academic and lay audiences through national and international conferences.

**Strengths and limitations of this study**

- The use of a coherent patient and public involvement strategy, which includes a parent of children with ID as a coinvestigator, a Parent Advisory Group comprising parents of children with and without ID and a Children and Young People (CYP) Advisory Group established through working in partnerships with schools whose pupil population includes those with ID.
- The use of traditional, creative and digital research methods will facilitate the inclusion of a wide range of participants, including CYP with ID, often described as a vulnerable population who are frequently excluded from research.
- Matching two groups of CYP, those with and without ID, will strengthen our ability to identify inequality where it exists and understand why it arises and for whom.
- The exclusion of parents who require an interpreter due to the added challenge this presents in gaining a thorough understanding of the needs of children with ID particularly those with communication difficulties.
- The restriction of only four sites for inclusion in phase 2 due to resource constraints, hence the inclusion of a robust process for selecting sites.

## Introduction

The preferred term for intellectual disability (ID) in the UK is learning disabilities. However, we use the term ID throughout the protocol as this is used consistently internationally.

It is widely recognised that people with ID have more health needs that often remain unmet than the general population. In 2007, Mencap, a UK charity, published ‘Death by indifference’^1^ detailing case histories of six people with ID who died in hospitals from avoidable conditions and calling on the government to take ‘serious action’. An independent inquiry into access to healthcare for people with ID followed, revealing significant system failures and reporting that patients with ID were treated less favourably than others, resulting in prolonged suffering and inappropriate care. The report of this inquiry, ‘Healthcare for All’,^2^ identified the invisibility of people with ID within health services, and the lack of priority given to identifying their particular health needs. Training and education about ID were found to be very limited. Combined with ignorance and fear, lack of training was identified as reinforcing ‘negative attitudes and values towards people with learning disabilities and their carers’ and ‘contributing significantly to a failure to deliver equal treatment, or to treat people with dignity or respect’. A need to strengthen the systems for assuring equity and quality of health services for people with ID at all levels was identified.

A confidential inquiry into premature deaths of people with ID (CIPOLD)^3^ including 14 children and young people (CYP) aged 4–17 followed. It emerged that in comparison with the general population, ‘more people with ID died from causes that were potentially amenable to change by good quality healthcare’. All aspects of care provision, planning, coordination and documentation were found to be significantly poorer for people with ID. A plethora of recommendations and guidelines are now available to support hospitals in ensuring that ‘people with ID are included as “equal citizens, with equal rights of access to equally effective treatment”’.^2^ Mencap has worked with healthcare professionals and Royal Colleges to develop the ‘Getting it Right Charter’^4^ highlighting key activities that all healthcare professionals should undertake to ensure that there is equal access to health, including the appointment of a learning disability liaison nurse (LDLN) in every hospital. While 200 trusts, hospitals and organisations have signed up to the Mencap Charter demonstrating their commitment to change, a current feasibility audit of adult ID care pathways found that only 56% of the nine acute trusts that took part had a liaison nurse in place.^5^ Providing reasonably adjusted services for people with ID is a legal requirement.^6^ Yet, the largest study of its kind to date^7^ found that the delivery of reasonable adjustments in the adult hospital setting was haphazard, with a lack of (1) effective systems for identifying patients with ID and (2) clear lines of responsibility for implementing reasonably adjusted care to individual patients.

The direct relevance that current recommendations about the care of ‘people’ with ID have to CYP, and guidance on the best way to implement them in the child health setting, are missing. The main thrust of initiatives aimed at reducing health inequalities faced by people with ID has been on improving access to healthcare among *adults* rather than the health inequalities faced by CYP.^8^ Hence, what we still do not know is the extent to which available recommendations *should* be applied to CYP with ID; to what extent they *are* being applied to CYP with ID or, if they are being applied, what *difference* they are making to patients, parents and staff.

### CYP with ID and their families

CYP with ID routinely experience particularly poor health outcomes. A review of the evidence on the prevalence and determinants of health conditions and impairments among CYP with ID in the UK^9^ found that the risk of children being reported by their main carer (usually their mother) to have fair/poor general health is 2.5–4.5 times greater for those with ID compared with their non-disabled peers,^8^ ^10^ a finding only partially accounted for by differences in socioeconomic status.^11^ As well as having intellectual impairment, these children may have sensory impairments and physical impairments, such as cerebral palsy,^12^ that adversely affect their speech, feeding and mobility. CYP with ID are also almost twice as likely to report three or more health problems and more than four times as likely to suffer from a psychiatric disorder than children without ID.^10^ ^13^ Increasing numbers are dependent on technological equipment for their survival.^14^

Children with disabilities experience more frequent and lengthier hospital admissions than children without disabilities^15^ and have contact with numerous professionals, often attending the same hospital many times in a week.^16^ They are also more likely than other children to be absent from school. In those with profound multiple learning difficulties, 62% of absences were accounted for by illness and 13% from attending medical/dental appointments.^11^ The ability for CYP with ID of *all* ages to understand information about hospital care and treatment will be limited, they may not be able to communicate their needs verbally, and may need additional support with all aspects of hospital life. While many CYP will find it hard to cope emotionally when they are in an unfamiliar hospital environment, those with ID who have challenging behaviour^17^ may find it particularly difficult.

Within the National Service Framework (NSF) for CYP in hospital^18^ the distinct service requirements of ‘disabled’ children are recognised, as is their greater need for personalised, child-centred care. However, the NSF framework precedes the latest evidence on the care of people with ID in hospital and may no longer be fit for purpose for meeting the specific intellectual, emotional, social and physical needs of CYP with ID. A number of children's hospitals have introduced nursing posts with a specific focus on improving care for CYP with ID but provision varies geographically and over time, and has not been formally evaluated. Many reports have highlighted the need to review National Health Service (NHS) services for *disabled* children and their families. The most consistent message is that services need to be tailored to meet the individual needs of these patients and it is imperative that their views are incorporated at every level of service delivery. This message applies equally, if not more so, to CYP with ID, whose struggle to get their views heard is widely recognised.

### Evidence of acceptability and effectiveness of services

Few researchers have focused on how acceptable and effective hospital services are in meeting the needs of CYP with ID and their families. More importantly, the voice of CYP with ID is largely non-existent. Conversely, there has been some research conducted with CYP without ID, including those with long-term conditions, to understand the hospital experience from their perspective.^19–23^ We know from this body of work the range of fears and anxieties that CYP express about being in hospital, as well as having some understanding of what supports them to feel safer, happier and more positive about their experience. What we do not know is whether CYP with ID have the same needs and experiences. A recent review of qualitative studies reporting on the experience of *disabled* children as inpatients^24^ led to the conclusion that their experience was ‘variable and not always optimal’ and that providing information would improve their experience. Importantly, of the eight studies included in this review, only two focused specifically on the care of children with ID and within these, only two individual children were interviewed. Of significance is that these two CYP, despite talking positively about nursing staff, were reported to be ‘less positive in general about their hospital stay than their parents’. Similarly, in a small Australian study^25^ exploring the views of four children with cerebral palsy about their experience of the medical consultation, it was reported that ‘whilst children and mothers had similar views about communication, there were obvious differences in what was perceived to be important’. Children described wanting to be included even if they did not understand what was being said, and expressed a desire to be informed of any tests or procedures before they happened, rather than having things ‘done’ to them. From this small body of evidence, we can draw three important conclusions, (1) evidence of what CYP with ID think about hospital and what they want from hospital services is lacking, (2) given the opportunity, some CYP with ID are able to share views about hospital and what is important and (3) CYP with ID do not necessarily view hospital in the same way as their parents. We know from our own experience and that of Sharkey *et al*^26^ that recruiting CYP with ID into research while they are in hospital can be challenging. However, this should in no way preclude their involvement.

A small body of qualitative research has been conducted with parents of CYP with ID to understand their own and their child's experience of hospitalisation^27^ ^28^ Avis and Reardon^27^ explored parents' perceptions of nursing care and attitudes and how their child's experience could be improved. They report parental feelings of stress, anxiety and fear, an expectation to care for their child, a lack of trust and confidence in staff and a lack of information and preparedness. Communication with staff was reported as the biggest issue that needed addressing. More recently Sharkey *et al*^26^ have reported on the barriers and facilitators to communicating with disabled children when inpatients. Interviews with parents and professionals revealed that ‘communication with disabled children on the ward was perceived as less than optimal’ and that ‘staff perceived time pressures and lack of priority given to communicating directly with the child as major barriers’. They found that parents could feel a ‘weight of responsibility’ concerning their child's communication that could make them reluctant to go home and leave their child alone. An in-depth qualitative study^29^ carried out by Oulton *et al* supports these findings. Parents described a sense of devoted protection towards their child with ID, which meant they were simply not willing to take any risks by leaving their child in the care of someone they did not have complete confidence in. Moreover, on the rare occasions when they felt they had no option but to leave their child, the occurrence of any problems could devastate trust in the overall system, with some refusing to access those particular services again. Ultimately, parents felt they had to take complete responsibility for their child's health and well-being, even in hospital. The general tone was one of apprehension that other care providers lacked the specialist knowledge they held about their child; anger that their advice was often ignored and concern that others did not share their dedicated commitment to their child. A feeling that professionals devalued them and their child with ID was also reported. More recent ethnographic research has revealed that meeting the specific non-medical needs of CYP with ID can present a challenge to hospital staff where the focus was on providing highly specialist, complex medical care for all its patients. Staff identified that having more time, resources and training would help them provide the individualised approach to care that these patients needed.^30^

## The current study

### Aims and objectives

Primary aims are as follows:

1. To identify the cross-organisation, organisational and individual factors in NHS hospitals that facilitate CYP with and without ID and their families receiving equal access to high-quality care and services.
2. To identify the cross-organisation, organisational and individual factors in NHS hospitals that prevent CYP with and without ID and their families receiving equal access to high-quality care and services.

Secondary aim is as follows:

- To develop guidance for NHS Trusts about the implementation for successful and effective measures to promote equal access for CYP with ID and their families.

#### Research questions

From the perspectives of the families and clinical staff:

1. Do CYP with and without ID and their families have equal access to high-quality hospital care that meets their particular needs?
2. Do CYP with and without ID, assisted by their families, have equal access to hospital appointments, investigations and treatments?
3. Are CYP with and without ID and their families equally involved as active partners in their treatment, care and services?
4. Are CYP with and without ID and their families equally satisfied with their hospital experience?
5. Are safety concerns for CYP with and without ID the same?
6. What are the examples of effective, replicable good practice for facilitating equal access to high-quality care and services for CYP with ID and their families at the study sites?
7. What indicators from the data and the literature suggest the findings may be generalisable to adults with ID and other CYP with long-term conditions in the hospital setting?

## Methods and analysis

### Theoretical/conceptual framework

**Figure 1:**
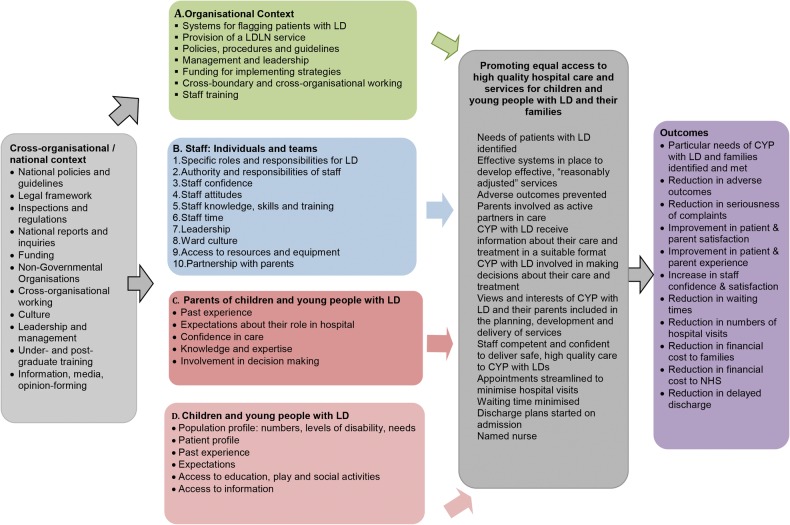
Theoretical framework.

This study takes a systematic approach to an empirical identification of the factors that affect access to high-quality hospital care for CYP with ID and their families. Building on the work of Tuffrey-Wijne *et al*,^31^ a theoretical framework for understanding the range of factors at the organisational and individual level that might impact on the delivery of hospital care to CYP with ID and their families has been described (figure 1). A synthesis of existing research, policy and guidelines and the team's expertise and research in the field of ID informed its development. Included are outcomes that might be associated with effective measures for promoting equal access. We intend to repopulate this framework with barriers and facilitators to promoting equal access to safe, high-quality hospital care for CYP with ID and their families identified through inductive analysis of data and by systematically testing the theoretical and empirical framework throughout this study.

### Design

A transformative, mixed methods case study design^32^ will be used. A ‘transformative’ case study is one that focuses on under-represented or marginalised populations, such as CYP with ID and their families. It involves being sensitive to the needs of this population and conducting research with the overall aim of improving social injustice. In terms of recruitment, our focus will be on avoiding stereotypical labels, recognising participant diversity and using sampling strategies that improve inclusiveness. We will work closely with sites to ensure that a diverse range of families are invited to take part and that a screening log is maintained, documenting any reasons for not providing eligible families with information about the study and reasons why participants decline where this information is available. With regards research methods, a transformative design prioritises those that give ‘a voice to the powerless and voiceless’^33^ and that are sensitive to the community's cultural context. Our combination of traditional, creative and digital research methods have been carefully selected on this basis, and will be individualised to each family and used flexibly in accordance with their needs and preferences. Using transformative research, the aim is to generate results that are useful to participants and credible to stakeholders and policymakers. Our overall aim is to identify inequality where it exists and understand what factors facilitate and prevent equality of healthcare for CYP with ID such that improvements can be made in the way that services are delivered. We believe that by getting it right for CYP with ID we can get it right for all CYP with long-term conditions.

**Figure 2:**
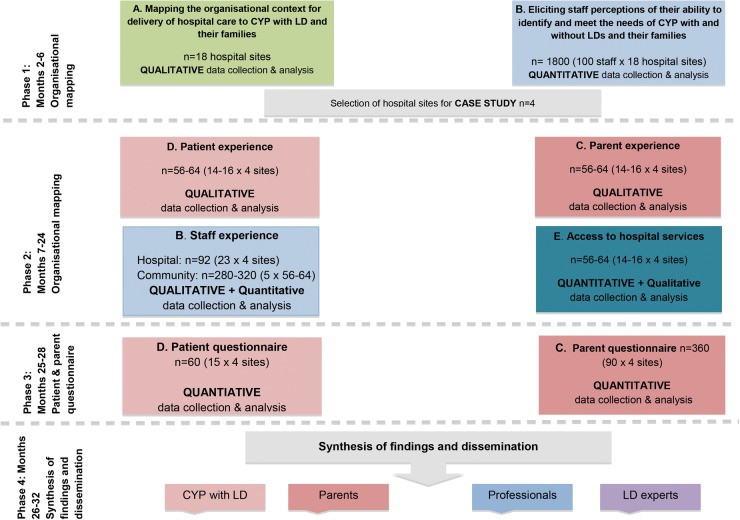
Phases of data collection.

**Figure 3:**
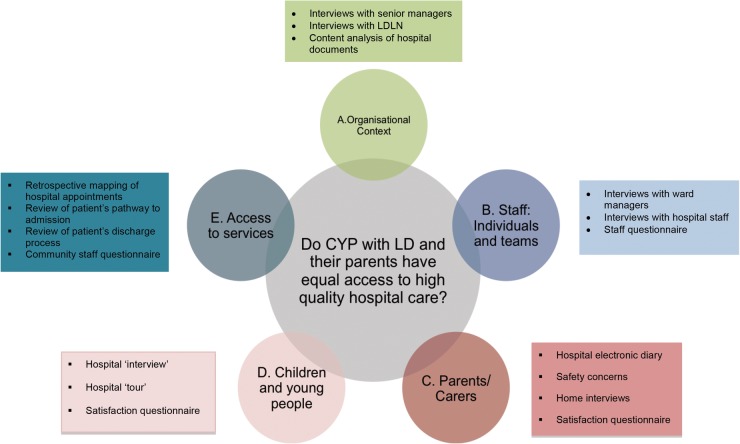
Strands of qualitative and quantitative data collection.

Case study design is ‘an empirical inquiry that investigates contemporary phenomena in depth and within its real-life context’.^34^ In this study, a single hospital site represents each case and four cases will be included. In each hospital, for every CYP with ID recruited, a CYP without ID will be recruited as a comparator case, thereby allowing the experience of the two groups of patients to be compared. This is a complex study, requiring data to be gathered consecutively in four distinct phases over 3 years (figure 2). Case study design is characterised by a convergence of diverse sources of quantitative and qualitative data (figure 3) and is therefore well suited to evaluating the multiple elements likely to shape and influence whether CYP with and without ID and their families receive equal access to high-quality hospital care and services. The production of rich descriptions of the phenomena through in-depth interviews and digital research methods will allow the many complexities of the situation and factors that can contribute to those complexities to emerge.^35^

### Sampling and recruitment

#### Phase 1: organisational mapping and staff questionnaire

All of the children's hospitals in England will be formally invited to take part in phase 1 via email through the Association of Chief Children's Nurses. We have estimated recruiting nine of these sites into the study. For each of the children's hospitals included, a second hospital in the same region, serving CYP with ID, will be recruited, giving a final sample of 18 hospitals. This sampling method will allow a range of specialist (children's hospitals) and non-specialist (district general, teaching) hospitals, in urban and rural locations to be included. To be eligible for selection, non-specialist hospitals must have at least two children's ward and be within reasonable distance of the children's hospital to aid data collection between the two sites. The sampling strategy for all phases is shown in table 1.

**Table 1:** Sample strategy and characteristics

Phase	Participants	Sampling strategy	Sample size
1	Senior managers/LDLN	Senior managers from the trust identified by the local collaborator as having relevant knowledge of hospital services and provision. All staff with a defined role for CYP with ID.	36–54
	Hospital staff	All clinical and non-clinical staff with contact with CYP and their families will be invited.	1800
2	CYP and parents	A purposive sampling strategy using a sampling matrix to ensure diversity according to level of ID, age, ethnicity.	56–64 CYP 56–128 Parents
	Hospital staff	All ward managers on each study ward will be invited. A purposive sample of hospital staff identified by parents or CYP as making a difference to their care.	12 ward managers 112–128 hospital staff
	Community staff	All community professionals named by parents as being involved in the care of their child.	280–320
3	CYP and parents	All CYP and parents discharged from participating wards.	60 CYP 360 Parents

CYP, children and young people; ID, intellectual disability; LDLN, learning disability liaison nurse.

#### Phase 2: case studies

Selection of hospital sites for phase 2 will be a four-step process:

1. *Assessing eligibility*: Hospital sites will only become eligible for phase 2 if they demonstrate accessibility to sufficient numbers of CYP with and without ID and good hospital engagement. Good hospital engagement will be assessed by the core research team on two criteria: (1) timely research and development approval and engagement from the named local collaborator, and (2) timely completion of data collection activities.
2. *Ensuring variability*: To ensure site variability in amount of ID provision, eligible hospitals will be grouped by the core research team according to whether they have a lot, a little, or no initiatives/appointments of an ID professional with a remit to improve care for CYP with ID.
3. *Designing scoring criteria*: Members of the Study Steering Committee will then be asked to design scoring criteria to enable objective selection of the sites for phase 2 based on:
  1. The strength of organisational context for delivery care to CYP with ID;
  2. Staff's perceived ability to identify and meet the needs of CYP with ID;
  3. Initiatives/appointments of an ID professional with a remit to improve care for CYP with ID.
4. *Applying scoring criteria*: The scoring criteria developed by the Study Steering Committee will be applied by the executive research team and sites will be selected on that basis.

Sites will be anonymised to prevent selection bias.

#### Operational definition of ID

The theoretical definition of ID is not always easily operationalised in practice. Among very young children, only severe ID is likely to be apparent^36^ and some CYP never receive a formal diagnosis of ID but remain categorised as having ‘developmental delay’ or a ‘syndrome without a name’. Hospital staff do not always know what is meant by ID or which CYP on their ward have this diagnosis. A CYP will be classified as having an ID if any ONE of the following is documented in the medical notes:

1. The CYP has an ID.
2. The CYP has a condition that is always accompanied by some degree of ID, for example, Down syndrome.
3. The CYP has global developmental delay (GDD) and they are aged over 10 years old.
4. The CYP attends a school for Children with Special Educational Needs and their parent confirms the child has an ID.

We have adopted a broad approach to defining ID because it is precisely those issues around the identification of this population that need exploring.

CYP with ID will be broadly matched with another CYP with a long-term condition. They will be matched on four criteria: (1) age, (2) number of comorbidities, (3) expected length of stay, (4) reason for admission. The aim is to recruit two samples of CYP with and without ID who are of similar age, with equal complexity of health needs and who are admitted to the same hospital during the study period.

### Inclusion and exclusion criteria

Table 2 summaries the inclusion and exclusion criteria for each of the participant groups.

**Table 2:** Inclusion and exclusion criteria

Participants	Inclusion criteria	Exclusion criteria
CYP with ID	Aged 4–18, known ID (as defined above) Expected minimum inpatient stay of 3 nights	Acute health problem only
CYP without ID	Aged 4–18 Expected minimum inpatient stay of 3 nights	Acute health problem only
Parents	Is able to speak English (phase 2 only) Is able to read English or one of five languages selected for translation (phase 3 only)	None
Hospital staff	Is involved in the care of one of the CYP recruited to the study	None
Community staff	Is attached to one of the recruiting wards	None

CYP, children and young people; ID, intellectual disability.

### Methods

#### Phase 1

##### Staff interviews (research questions 1–7)

Interviews with senior managers and LDLN will be semistructured and conducted face-to-face or via telephone. The focus of interviews will be on the delivery of services to CYP with ID at the organisational level.

##### Content analysis of hospital documents (primary aim)

Hospital documents will be collected electronically and a content analysis conducted. The following documents will be included: Communication Policy, Admission and Discharge Policy, Complaints Policy, Child Protection Policy, the latest Patient Experience/Satisfaction Surveys and any specific ID Policy. A search and find exercise using predefined terminology (ie, learning disability, special needs, intellectual disability) will be used to ascertain references to CYP with ID and a thematic framework will be created based on content. The first set will be examined in detail and a simple coding frame developed for subsequent documents.

##### Staff questionnaire (research questions 1–3)

The staff questionnaire has been devised to elicit staff perceptions of their ability to identify the needs of CYP with and without ID and their families and provide high-quality care to effectively meet these needs. The questionnaire will focus on six key areas: staff knowledge, skills, training, confidence, time and resources. The questionnaire will be piloted to ensure it is acceptable and relevant to staff.

#### Phase 2

##### Interviews with CYP (research questions 1–3)

The Mosaic approach,^37^ ^38^ combining the ‘traditional methodology of observation and interviewing with the introduction of participatory tools’^37^ will be used to guide interviews with CYP. The aim is to have a toolkit of creative and digital techniques available that draw on each individual's strengths, thereby enabling them to share their experience and preferences in whatever way they are able and comfortable with. The primary method of data collection will be ‘Talking Mats’, a communication symbols tool consisting of a pictorial framework based on three sets of picture symbols—issues relevant to the topic, factors relating to each issue and emotions to allow participants to indicate feelings about each factor. The method is suitable for CYP of all ages and communication abilities and can therefore be offered to all participants irrespective of whether they have an ID. Arts-based activities, photography and a hospital tour^39–42^ are other ways that CYP will be able to share their views. Data collection sessions will take place in a quiet room on or close to the ward, depending on each CYP's personal preference and health needs. Some CYP, including those with ID, may find it difficult concentrating for long periods of time and in these circumstances a few short sessions may be preferred to one longer session. CYP and parents will guide the researcher as to what would be most appropriate. Young people's preference for their parent(s) to be present or absent during the sessions will be respected.

##### Parent electronic diary (research questions 1–3)

Parents will be given an android ‘tablet’ (password-protected and security-tagged) and invited to complete a hospital diary during their child's inpatient admission. This will be preinstalled with a virtual notebook for simply and instantly uploading audio and video files, photographs and written comments. Parents will be encouraged to document their thoughts and feelings in relation to key events during their hospital stay such as admission, discharge and their child's investigations and treatments. Parents will have a choice about whether and when to share uploads, thereby giving them control about what becomes data. We know that parents can be reluctant to leave their child to be interviewed, even for short periods—an electronic diary offers flexibility in how they tell their story and can be completed at any time of the day/night. By incorporating the use of novel, digital research methods, we aim to give parents flexibility and enhance the findings through the capturing of ‘live data’. Parents will also be offered a paper diary as an alternative to the ‘tablet’.

##### Home interviews with parents (research questions 1–3, 5)

Home interviews will be conducted with parents as soon as possible after discharge from hospital, preferably once the child/young person has returned to school. The interview guide will focus on parents' experience of accessing and using hospital care and services for themselves and their child. Data recorded on the parent diary will be used as a further prompt. Questions about the child's pathway to admission and their experience of discharge will be included. Parents will also be asked to identify up to five staff who made a ‘difference’ (positive or negative) during their child's admission, one to two of whom will be invited for interview. Details of community professionals in contact with their child will also be collected. Parent interviews are expected to last 1–2 hours.

##### Interviews with hospital staff (research questions 1–3, 5, 7)

Semistructured interviews with hospital staff will be conducted face-to-face or by telephone. They are expected to last 30–60 min. Flexibility will be provided as to the timing and location of interviews to minimise staff burden.

##### Completion of the ‘daily safety reporting tool’ (research question 5)

In light of qualitative evidence that parents of CYP with ID can lack confidence that their child is receiving high-quality hospital care and subsequently feel responsible for monitoring their care, parents will be asked to complete an adapted version of the daily safety reporting tool^43^—a six-item tool which asks parents to identify their safety concerns in terms of: medication, communication and information, equipment, unexpected complications of care, hygiene/cleanliness and other safety problems. Completion of the tool will enable perceptions of safety between the two groups of parents to be compared. Information collected will be used as a prompt during home interviews.

##### Retrospective mapping of hospital appointments (research question 2)

For each CYP, a retrospective mapping will be conducted of all inpatient stays and outpatient appointments for the previous 2 years using the electronic hospital appointment system to retrieve a range of data (table 3).

**Table 3:** Data for retrospective mapping exercise

Inpatient admissions	Outpatient appointments
Age of patient	Age of patient
Diagnosis	Diagnosis
Date of admission	Date of appointment
Admitting ward	Time of appointment
Admitting team	Admitting team
Reason for admission	‘Did not attend’ status
Anticipated date of discharge	Reason for ‘Did not attend’ status
Date of discharge	
Discharge location	

##### Questionnaires by community-based professionals (research questions 1–3)

Community professionals named by parents as being involved in the care of their child will be sent an anonymised questionnaire in the post, with a stamped address return envelope. The questionnaire will be a modified version of the hospital staff questionnaire from phase 1 with a particular focus on access to secondary and tertiary care for CYP with and without ID.

All interviews conducted during phase 1 and phase 2 will be recorded and transcribed verbatim with participant's permission.

#### Phase 3

##### Patient and parent satisfaction questionnaire (research question 4)

There is a lack of validated patient/parent satisfaction questionnaires, particularly for CYP and those with ID. Drawing on the best available tools (http://www.chimat.org.uk/default.aspx), a questionnaire will be purposefully designed to answer the research question. Multiple versions of the questionnaire will be developed for CYP across the age range and with differing levels of cognitive functioning. Questionnaires will be piloted with a group of CYP with and without ID and their parents beforehand. A sealed box will be available on the ward for participants to leave their completed questionnaire prior to discharge and free post envelopes will also be available for return by post.

#### Phase 4

##### Dissemination workshop

A workshop will be held towards the end of the study for CYP, parents, professionals and experts in the field of ID to disseminate findings and decide the content of a DVD and/or training package that will be used in practice to inform students and staff about the barriers and facilitators to the delivery of high-quality care for CYP with ID and their families.

### Data analysis

A model for mixed methods data analysis^1^ will be used. Qualitative and quantitative data will be analysed within each phase using appropriate methods before merging and connecting them through a period of data synthesis. During data synthesis, the research team will use quantitative data to explain and illustrate qualitative findings, and look for congruence and incongruence between qualitative and quantitative findings. In particular, the team will look for instances where there is incongruence between policy and practice, using specific queries within the NVivo programme to address these issues and explain any incongruence. It is at the stage of data synthesis that barriers and facilitators to ensuring CYP with ID and their families receive equal access to high-quality hospital care and services will be highlighted, looking for specific examples of successful and effective measures that promote equal access. The final analytical framework will be compared with our theoretical framework and the initial common analytical framework, in order to generate a final empirical framework of factors that affect the promoting of equal access to high-quality hospital care for CYP with and without ID and their families.

### Qualitative

Multiple sets of qualitative data will be generated from this study that are best analysed inductively using the framework method. This matrix based analytic method facilitates rigorous and transparent data management ‘such that all the stages involved in the “analytic hierarchy” can be systematically conducted’.^44^ The method involves five distinct, but highly interconnected stages: familiarisation; identifying a thematic framework; indexing; charting; mapping and interpretation. The strength of using framework is that it allows easy access to the synthesised data so that it can be continually revisited, which is important when conducting multicentred, mixed methods research over four phases. The approach enables data to be examined within cases across a range of different themes, thereby facilitating comparisons to be made between and within case study sites. Furthermore, the process is well suited to research involving group-level and individual-level analysis. The data will be managed using NVivo, a qualitative data analysis programme.

### Quantitative

#### Separate quantitative analyses will analyse

1. *Hospital staff questionnaire data (phase 1)*. Analyses will follow previous studies of staff questionnaires of patients with ID in hospitals.^7^ Descriptive comparisons for each of the six key areas of the questionnaire between responses pertaining to CYP with and without ID will be presented (eg, frequencies, percentages, means and SDs, medians and IQRs). Comparisons will also be presented for subgroups of respondents categorised by staff group (eg, doctors, nurses, professions allied to medicine, non-clinical staff), staff grade and site.
2. ‘*Safety concerns*’ *data using the daily safety reporting tool (phase 2)*. Number and type of safety concerns will be compared and analysed descriptively.
3. *Community-based staff questionnaire (phase 3)*. Responses will be compared descriptively and analysed in the same way as for the hospital staff data, described above in 1.
4. *Parent and patient satisfaction with hospital care (phase 3).* Responses to this questionnaire will be compared descriptively and analysed in the same way as for the hospital staff data, described above in 1.

## Ethics and dissemination

This study includes data collection involving vulnerable CYP. The research team has long-standing expertise in conducting research in sensitive areas. A range of steps will be taken in order to safeguard all informants from undue harm in accordance with the principle of beneficence. We will pay particular attention to obtaining assent/consent from research participants with ID, using a range of accessible study information materials combining words, pictures and symbols as well as a talking photo album. A model of individualised assent, developed in line with the latest guidance from the Nuffield Council on Bioethics,^45^ will be used to ascertain whether CYP are able to say what they think about the research and to make an independent decision about taking part. We will pay particular attention to the various ways in which CYP may express their wish to withdraw from the study and their response to the ending of the research relationship. An awareness of the issues associated with collecting data in the hospital setting is important to minimise risks to participants, for example, where children may be too unwell to take part in data collection activities or under infection control restrictions, being otherwise occupied with tests and treatments or being overheard by other patients and staff. A particular ethical issue associated with case study research is maintaining participant confidentiality. While it is impossible to prevent staff from knowing that a family is taking part because data collection is taking place on the ward, strict coding and anonymisation procedures will be used to ensure their data remain confidential. When publishing results, care will be taken not to report information that will enable research sites or individuals to be identified, for example, in relation to rare conditions, provision of rare treatments or geographical location.

The primary output will be guidance for commissioners and providers of NHS hospital services for CYP with ID and their families. Following synthesis of the findings and the dissemination workshop, the Executive Research Team will consult widely with members of the Steering Committee, Parent and CYP Advisory Groups about the content and format of guidance document and the wider implementation strategy. We will engage with the Association of Chief Children's Nurses and the senior management from all phase 1 sites, as well as professional bodies such as Royal College of Paediatrics and Child Health (RCPCH) and relevant third-sector organisations such as the British Institute of Learning Disabilities (BILD).

We will disseminate the results of the study through international peer-reviewed journals and national and international conferences. We will develop a social media strategy to ensure ongoing dissemination of findings and user engagement throughout the project, and to build a network/community of interested users/stakeholders. A report of the study findings will be sent to participants in a range of accessible formats.
